# A New Viewpoint on the Etiopathogenesis of Depression: Insights From the Neurophysiology of Deep Brain Stimulation in Parkinson's Disease and Treatment-Resistant Depression

**DOI:** 10.3389/fpsyt.2021.607339

**Published:** 2021-04-09

**Authors:** Amílcar Silva-dos-Santos, Miguel Sales, Ana Sebastião, Ricardo Gusmão

**Affiliations:** ^1^NOVA Medical School (NMS/FCM) – NOVA University of Lisbon, Lisbon, Portugal; ^2^Department of Psychiatry – Hospital Vila Franca de Xira, Vila Franca de Xira, Portugal; ^3^Faculty of Medicine and Unit of Neurosciences, Institute of Pharmacology and Neurosciences, Institute of Molecular Medicine, University of Lisbon, Lisbon, Portugal; ^4^EPI Unit, Public Health Institute, University of Porto, Porto, Portugal; ^5^Departamento de Ciências da Saúde Pública e Forenses, e Educação Médica, Faculdade de Medicina da Universidade do Porto, Porto, Portugal

**Keywords:** deep brain stimulation, etiopathogenesis, Parkinson's disease, treatment-resistant depression, depression, mental kindling-like mechanism

Journalist:

Aren't you afraid of not feeling human after the implantation of

the deep brain stimulation electrodes to treat your resistant depression?

Patient:

When I was suffering from treatment-resistant depression I was not feeling human!

*(A patient of Dr. Helen Mayberg treated with Deep Brain Stimulation)*.

## Introduction

In this opinion article, we humbly propose a new viewpoint on the etiopathogenesis of depression. We base the model on our interpretation of selected recent neurophysiological findings, mainly regarding Deep Brain Stimulation (DBS) to treat Parkinson's Disease and also DBS to treat Treatment-Resistant Depression (TRD). We coin the idea a *mental kindling-like mechanism*. However, future clinical research, possibly complemented with human laboratory research or animal experiments, will be needed to test the validity of our proposed model. On the current manuscript framework, following the first and introductory section, we will set the stage by presenting selected studies and insights mainly from DBS to treat TRD and PD, on the second and middle section. Finally, we will discuss the proposed viewpoint on the third and last section of the present work.

## Selected Neurophysiological Studies and Insights From DBS to Treat Treatment-Resistant Depression and Parkinson's Disease

### DBS as a Therapy for PD

DBS was first introduced to medical practice by Benabid et al. (1). They discovered that high-frequency stimulation of the thalamus diminished tremor. Later, guided by the neurophysiological studies of Bergman et al. on the neural circuits involved in PD (2), Benabid stimulated the subthalamic nucleus with high-frequency current and achieved significant improvement of PD symptoms (3). PD has been linked to degeneration of dopaminergic neurons in the substantia nigra (4), which eventually results in an abnormal neuronal activity in the basal ganglia circuitry. This abnormal activity is characterized by synchronous bursts, which resemble epileptiform activity (5). These abnormal bursts affect the activity in the thalamus and cortical areas, causing the symptoms of PD (4). DBS has a therapeutic effect on PD because it disrupts the abnormal bursting in the basal ganglia—corticothalamic loops (6).

### DBS as a Therapy for Depression

In 2005, Mayberg's group reported that DBS was effective for the treatment of patients with depression who were resistant to medication and/or electroconvulsive therapy (ECT) (7). The idea of such treatment was based on neuroimaging studies that revealed, in some patients with depression, abnormal hyperactivity of the subcallosal cingulate cortex (SCC) also known by area 25 of Broadman (BA25) or subgenual cingulated (Cg25). Mayberg et al. hypothesized that this hyperactivity detected in neuroimaging studies could be reduced by DBS. Indeed, DBS of the area 25 reduced depression symptoms in patients with abnormal neural activity (7). After this work, other studies have been published regarding the stimulation of area 25 of Broadman in depression making this area the most targeted in DBS for treatment-resistant depression (7–12). The lateral habenula is another area targeted in depression. It has been reported that the hyperactivity in this area can be ameliorated with DBS (13). Contrarily to the hyperactivation in the area 25 of Broadman and in the lateral habenula, there is hypoactivity in the nucleus accumbens (NAcc) that can be reverted by DBS in patients with treatment-resistant depression (14, 15). Other areas that have been stimulated in DBS are the ventral capsule and ventral striatum (16, 17), the internal capsule (18), the medial forebrain bundle (19), and the inferior thalamic peduncle (20). As it can be noted, there are different areas with distinct type of neural activity (hypo/hyperactivity) in treatment-resistant depression and DBS with high frequency stimulation can bring those activity to normal.

### DBS Mechanisms

The exact mechanisms by which DBS works are not fully understood. In particular, it may seem paradoxical that DBS inhibits neural activity in the stimulated area instead of stimulating the neurons, therefore acting in a similar way to an ablation (6). One explanation for the inhibitory effect of DBS is that in the thalamus it induces a release of inhibitory neurotransmitters such as the homeostatic neuromodulator adenosine that causes a decrease in the excitatory neurotransmission and a reduction of tremor via Adenosine A1 receptors activation (21, 22). Inhibition is not the only mechanism by which DBS alleviates symptoms of PD and depression. Other mechanisms have been suggested and reviewed elsewhere (23), such as replacement of pathological bursting by neuronal patterns driven by DBS (24).

The correction of abnormal brain activity is another proposed DBS mechanism of action. Since, on the one hand, there is abnormal brain oscillation in PD (25) that is similar to the neural activity observed in some models of seizures and epilepsy (26, 27), and on the other hand, it has also been reported abnormal brain oscillation between some areas in depressive animals, it was considered that, at least in part, some abnormal epileptiform oscillation might be an electrophysiologic trait of some neurologic and psychiatric disorders. The origin of some abnormal brain oscillations is at the core of our proposed *mental kindling-like mechanism* and it will be addressed in the third part of the current manuscript.

### DBS Works by Stimulating a Specific Brain Area. Why Do Therapies Such as Electroconvulsive Therapy (ECT) and Repetitive Transcranial Magnetic Stimulation (rTMS) Do Not Target a Specific Brain Area?

DBS of the subcallosal cingulated cortex (SCC) is effective in the treatment of depression. However, a patient-personalized protocol must be applied in order to be effective. Neuroimaging and electrophysiological studies during DBS surgery allow the determination of the exact and most adequate area to stimulate. It seems that SCC resembles a crossroad of different projections and the best effect is achieved by stimulating the exact crossing point (28). DBS targets specific areas that are functionally impaired in Depression. Some therapies such as the well-known ECT and the more recent TMS (initially applied to treat depression and then extended to other psychiatric disorders) (29–31), act in broader way. The duration of the effect varies among the different approaches: DBS only works when the stimulation is on, ECT's effects are more sustained (although requiring maintenance sessions), while TMS requires multiple sessions during weeks before achieving clinical results. The reason for these discrepancies is not known. A possible explanation could be that unlike DBS, that is more efficient locally, the other techniques could simultaneously modulate several regions and be more effective targeting the systemic/circuitries changes or, at least in part, the broad stimulation can reaches the more deep and specific areas through white matters pathways.

### Other Selected Neuroanatomical and Neurophysiological Findings Relevant to Our Viewpoint

**Figure 1:**
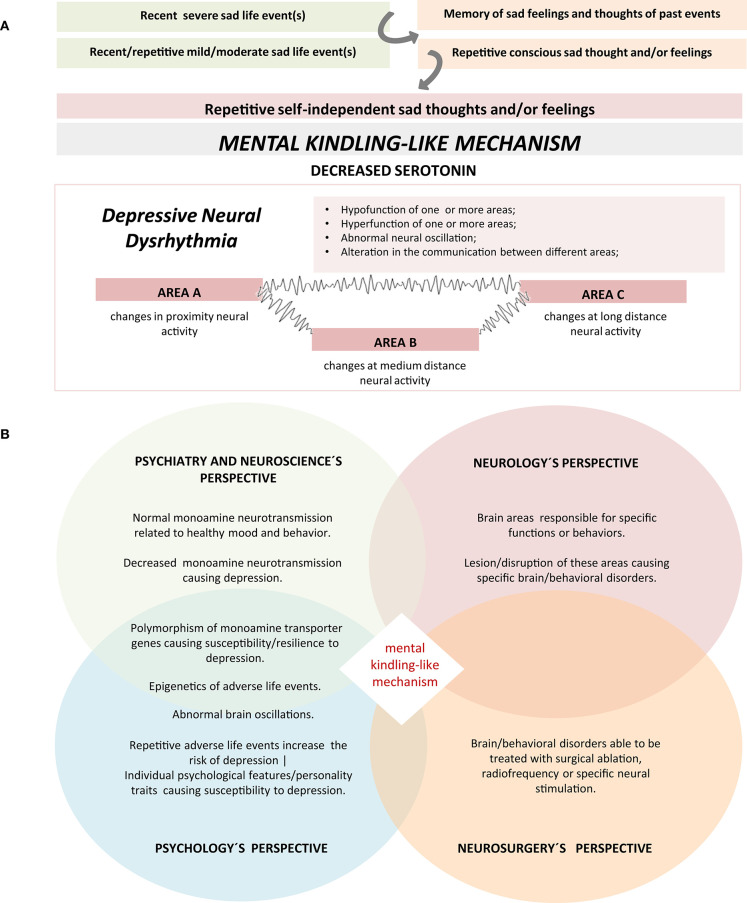
**(A)** A schematic of the hypothesis of the mental kindling-like mechanism in the etiopathogenesis of Depression. Repetitive sad life events, whether mild, moderate, or severe cause not only repetitive conscious thoughts and/or feelings, but also recall memories of sad feelings and thoughts. This pattern starts as a conscious process but can later become an independent or involuntarily process that can self-perpetuate and eventually cause the *mental kindling-like mechanism*. The mechanism may cause the depletion of serotonin and/or work as a trigger to dysregulate the neural nucleus and networks of emotion, resulting in the hypofunction/hyperfunction of different areas as well as abnormal communication between areas. **(B)** An hypothetical integrative physiopathological perspective of treatment-resistant major depression. A change in the neuronal activity of a brain region associated with depressive symptoms, decreased monoamine neurotransmission and abnormal neural activity caused, at least in part, by a possible *mental kindling-like mechanism* triggered by repetitive adverse live events in patients with genetic and/or psychological predisposition to depression. Due to the complexity and the multifactorial causes of depression, a single perspectives are not enough to understand the mechanisms of this disorder. A global and a more integrative perspective with the contribution of different disciplines can help understanding the mechanisms of depression.

Psychiatric or mental disorders are some of the most difficult diseases to understand. Unlike the diseases that can be explained by a single gene mutation, specific lesions or microorganisms, mental disorders usually have multiple causes. As it has been stated in this paper, there are several areas with distinct neural activities (hypo/hyperactivity) that can be targeted in DBS for depression. Besides the local abnormal neural activity observed in neuroimaging techniques, there are abnormal oscillations between different regions that can be recorded with non-invasive electrophysiological tools and that might be used as potential biomarkers to evaluate neuromodulation success (32). To simplify our perspective about depression, we will focus on a particular brain area, but we consider that the mechanism we propose can, possibly, be applied to different areas of the brain. Several studies suggest that the chronic hyperactivation of area 25 is one of the neural mechanisms of depression. Functional neuroanatomy studies demonstrated that simply thinking about sad life events causes hyperactivity of the area 25 (33). This hyperactivity can be reduced by several types of treatment: selective serotonin reuptake inhibitor (8), transcranial magnetic stimulation (TMS) (34) and electroconvulsive therapy (ECT) (35). Several studies reported that depression is a multifactorial disease. From a pragmatic point of view, in psychiatry we observe a frequent pattern in clinical practice: a typical depressive patient has familiar history of depression or other mental illness, and has experienced one or few severe life stress, or accumulated many mild/moderate adverse life events during weeks, months to few years before the depressive episode. A typical sequence that triggers depression is: first the patient voluntarily thinks about the stressors, but later this remembrance became automatic or involuntarily (independent of the patient's will) and eventually the patient becomes clinically depressed. It remains unknown how the psychological, environmental and biological/genetic factors interact together with a cognitive style to cause a chronic hyperactivation of area 25. It has been reported that there is abnormal brain oscillations in PD such as low-frequency rhythmic bursting in the basal ganglia (5, 25, 36) that are similar to epileptic activity. It has also been reported abnormal brain oscillations between some areas in depressive animals (37). Furthermore, it was suggested that this epileptiphorm abnormal oscillation can be an electrophysiologic trait of several neurologic and psychiatric disorders (26). A possible phenomena that we call *mental kindling-like mechanism* (Figure 1A) may cause such abnormal brain oscillation in some types of depression associated with significant live events and/or stressors.

## Discussion

According to our interpretation of the studies presented above, we propose that a severe adverse life event, or repetitive mild/moderate chronic negative life events can initially lead, at least in part, to conscious and transient hyperactivation of area 25 and then an automatic/involuntary processing of this information contribute to its chronic hyperactivation. From a clinical perspective, depressive patients often have constant, repetitive, involuntary thoughts that they not regularly pay attention to. They also say that recent sad life events triggers the memories of previous sad thoughts and feelings, which amplify the currents ones. Often, they recognize this pattern if the clinician asks them what are the content of their thoughts and feeling, at night when they are in the bed awaiting to fall asleep, or when they are not occupied with day to day tasks. Similarly to the kindling experimental model of epilepsy (38), in which repetitive external electric brain insults lead to abnormal automatic activity and hence to seizures/epilepsy, repetitive exposure to sad thought can lead to self-independent chronic abnormal brain activity and hence depression (abnormal brain epileptiform-like activity triggered by repetitive automatic or self-amplifying thoughts in the areas linked to emotions). However, some patients exposed to several life stressors do not develop symptoms of depression. This might be related to a resilience effect. As an example, Caspi et al. found that patients with the long allele of the serotonin transporter (causing better serotoninergic activity) are more resilient to stressful life events and hence less susceptible to develop depression than patient with the short allele of the same gene (39). In the context, we can speculate that *life events triggered kindling-like mechanism* or *mental kindling-like mechanism* will disrupt the neural activity, in susceptible patients, either locally in some brain areas (such as the 25) or more broadly in other emotion circuitries. According to this perspective to understand depression, the final endpoint of depression could be explained as a brain dysrhythmia (*depressive neural dysrhythmia*), either locally (one nucleus) or systemically (circuitries, different nuclei), in which genetic and neurochemical mechanism would play important roles. This perspective that we call *integrative perspective to understand mental disorders* (Figure 1B) can integrate different scientific points of view to understand psychiatric disorders: (1) Neurosurgery—functional neurosurgery, to ablate the abnormal area (excision, ablation, applying radiofrequency or the new electric stimulation approach); (2) Neurology: specific brain areas have specific functions. Some brain areas are involved in healthy emotions. A lesion on these areas could cause depression; (3) Psychiatry and Neuroscience: Monoamine theory. Decreased serotonin and/or noradrenalin neurotransmission can cause depression. Treatment of depression can be achieved by restoring the monoamine neurotransmission, epigenetic, and changes in brain oscillation. Also specific personalities have specific cognitive styles that confers susceptibility to specific disorders (ex. patients with ruminative tendency are prone to depression); (4) Psychology/psychotherapy: Repetitive mild/moderate life traumas or severe life trauma can increase the risk of depression. Applying a new psychological style can help to address and deal with specific adverse life events.

Although we formulated our opinion based on the interpretation of recent and relevant scientific data and clinical studies, we acknowledge that future research will be needed to test the validity of our proposed model.

## Author Contributions

AS-d-S wrote the first draft. All the authors edited and reviewed the manuscript.

## Conflict of Interest

The authors declare that the research was conducted in the absence of any commercial or financial relationships that could be construed as a potential conflict of interest.
